# A Full-Length Reference Floral Transcriptome of *Boehmeria tricuspis* Provides Insights into Apomeiosis and Polyploidy

**DOI:** 10.1155/2019/4025747

**Published:** 2019-12-16

**Authors:** Qing Tang, Ying Xu, Canhui Deng, Chaohua Cheng, Zhigang Dai, Zemao Yang, Chan Liu, Jianguang Su

**Affiliations:** ^1^Institute of Bast Fiber Crops, Chinese Academy of Agricultural Sciences, Changsha, 410205 Hunan, China; ^2^Key Laboratory of Biology and Processing of Bast Fiber, Ministry of Agriculture and Rural Affairs, Changsha, 410205 Hunan, China

## Abstract

*Boehmeria tricuspis* (Hance) Makino constitutes a hardy herbaceous or shrubby perennial native to East Asia that includes different ploidy levels and reproductive modes (diplosporous to sexual). Although several apomeiosis-associated genes have been described, the genetic control and molecular mechanisms underlying apomeiosis remain poorly understood. Moreover, the basis of the correlation between polyploidy and apomixis has not yet been clarified. We utilized long-read sequencing to produce a full-length reference floral transcriptome of *B*. *tricuspis*. Based on the generated database, gene expression of the female flowers of different ploidy levels and reproductive mode cytotypes was compared. Overall, 1,387 genes related to apomeiosis, 217 genes related to ploidy, and 9 genes associated with both apomixis and ploidy were identified. Gene Ontology analyses of this set of transcripts indicated reproductive genes, especially those related to “cell differentiation” and “cell cycle process,” as significant factors regulating apomeiosis. Furthermore, our results suggested that different expressions of stress response genes might be important in the preparation for apomeiosis transition. In addition, our observations indicated that the expression of apomeiosis may not depend on polyploidy but rather on deregulation of the sexual pathway in *B*. *tricuspis*.

## 1. Introduction

In botany, apomixis is defined as an asexual mode of reproduction through seeds [1]. Apomictically produced offspring inherit the genes of the mother only. In particular, gametophytic apomixis involves the combination of three major developmental steps: formation of a fully developed and unreduced embryo sac from a nucellar cell (apospory) or from a megaspore mother cell (diplospory) avoiding meiosis, development of a functional embryo from an unfertilized egg cell (parthenogenesis), and formation of functional endosperm either autonomously or following fertilization (pseudogamy) [2, 3]. The circumvention of meiosis (i.e., apomeiosis) constitutes the initial, fundamental step of apomixis. Without apomeiosis, parthenogenesis of reduced egg cells cannot result in exact replicas of the mother plant as they are produced from meiotic megaspores [4]. In addition, such haploid development hardly ever occurs due to lethality. Thus, understanding the genetic control and the molecular mechanisms underlying apomeiosis is critical for the comprehension of apomixis as a whole.

In plants, apomixis is almost exclusively associated with polyploidy [5–7] and has previously been considered a major consequence of genome duplication [3, 8, 9]. In fact, polyploidy constitutes an important and widespread genomic feature, especially among plants [10–12]. The gene redundancy and increased number of potential mutation sites associated with polyploidization provide the potential for rapid changes in the chromosome structure, gene expression, transposon activation, and developmental traits such as fertility, hybrid vigour, and apomixis [13, 14]. Based on the strong correlation with apomixis, polyploidy has been proposed as a mechanism by which sexual developmental pathways are deregulated following an increase in duplicated genes [8]. However, polyploidization is not a prerequisite for apomixis, as in the genus *Boechera* [15], in *Ranunculus kuepferi* [16], and in various *Paspalum* species [17]; natural apomictic diploids are observed. In addition, the polyploid nature of apomicts provides a challenge for genetic and genomic analyses [18]. For example, in *Poa pratensis* [19], *Paspalum notatum* [20, 21], and *Eragrostis curvula* [22, 23], the association between apomixis and polyploidy has been reported with some candidate genes related to ploidy and/or apomixis having been identified. However, the basis of the close correlation between polyploidy and apomixis has not yet been clarified; toward this end, the comparative expression analysis of apomictic and sexual cytotypes with differing ploidy levels might be of value.

*Boehmeria tricuspis* (Hance) Makino constitutes a robust herbaceous or subshrubby perennial that is distributed all around East Asia, mostly along the Yangtze River in China. *B*. *tricuspis* is monoecious or gynoecious, and the unisexual inflorescences originate from axillary positions among the leaves. The monoecious individuals have both male and female flowers, but the gynoecious ones only have female flowers. *B*. *tricuspis* shares a basic chromosome number of *x* = 14 [24], and there are several cytotypes at different ploidy levels (2*n* = 28, 42, and 56) in the wild. *B*. *tricuspis* belongs to the nettle family, Urticaceae, which also includes the well-known fiber crop (ramie), *Boehmeria nivea* (L.) Gaudich. *B*. *tricuspis* exhibits numerous characteristics such as simple growth requirements and high seed yield that render it both an excellent and intriguing system for the study of apomixis. In a prior research, we identified that the *B*. *tricuspis* triploid cytotype reproduces through obligate diplosporous apomixis, whereas diploids appeared to be a fully sexual cytotype [25]. Notably, at the functional megaspore-formed stage, in sexual ovules, the megaspore mother cell (MMC) produces four megaspores through meiosis, and one of the megaspores is selected to become a functional megaspore while the other ones degenerate, whereas in diplosporous ovules, MMC directly develops into a functional megaspore without the meiotic and degenerative process. Furthermore, we used second-generation sequencing platforms to perform a comparative transcriptome analysis of the diploid and triploid cytotypes of *B*. *tricuspis* and uncovered several functional terms possibly related to apomictic development [26].

Next-generation sequencing (NGS) technologies have been effectively utilized in the field of apomixis research to investigate those variations between sexual and related apomictic cytotypes [27–29]. Prior studies generated a reference transcriptome via second-generation sequencing (SGS) and reconstructed the transcriptome through the de novo assembly guided by homology to available reference genomes. Such processes are difficult for long transcripts, thereby preventing the accurate assembly of full-length (FL) transcripts in nonmodel species [30]. Moreover, in some cases, uncertain annotation accuracy may result as a consequence of the low-quality sequences generated by short-read RNA-Seq [31]. In comparison, long-read sequencing via third-generation sequencing (TGS) platforms (e.g., PacBio RS and Sequel) has recently become available and used to obtain FL transcripts to solve all problems specific to short-read technologies [32–34]. To overcome the problem of the relatively high error rate of TGS, a hybrid sequencing approach combining SGS and TGS technologies has been designed, which could provide high-quality and more complete assemblies in transcriptome studies [35–37].

Accordingly, in this study, we used the hybrid sequencing approach to produce a FL reference transcriptome of *B*. *tricuspis*. Based on the long-read databases obtained through PacBio Isoform sequencing (Iso-Seq), we compared transcriptomic profiles of the flowers of apomictic and sexual cytotypes with different ploidy in *B*. *tricuspis*. The objective of this study was to identify and functionally characterize genes possibly involving in the regulation of the expression of apomeiosis and showing ploidy-dependent expression. Furthermore, we aim to find the answers to the questions: (1) Is there strict correlation between polyploidy and apomixis in *B*. *tricuspis*? (2) What is the basis of the correlation between polyploidy and apomeiosis in *B*. *tricuspis*?

## 2. Materials and Methods

### 2.1. Plant Material and RNA Extraction

Two cytotypes of *B*. *tricuspis* previously reported [24, 25] were used in this work: the sexual cytotype “JG1” (2*n* = 2*x* = 28) and the full apomictic cytotype “ZJJ” (2*n* = 3*x* = 42). JG2 and HD were collected from Hubei Province and Zhejiang Province of China, respectively, and the ploidy levels were measured by chromosome counting in root tips as proposed by Bennett and Smith [38]. The four natural cytotypes were planted in the experimental field of the Institute of Bast Fiber Crops, Chinese Academy of Agricultural Sciences, Changsha, Hunan, China. With providing the cytoembryological observation of sexual and apomictic individuals of *B*. *tricuspis* [25], a calendar of the reproductive development was established ([Supplementary-material supplementary-material-1]). The MMC developmental stage could be estimated by the outer appearance of the flowers. Healthy female flowers at different developmental stages (MMC-formed, functional megaspore-formed, mature embryo sac formation, and mature embryo formation) were collected and immediately frozen using liquid nitrogen and stored at −80°C until use. The samples were harvested from three biological replicates of each cytotype at the same time of the day (10 am). For flower samples, total RNA was isolated using TRI Reagent (Sigma Life Science, St. Louis, MO, USA) according to the manufacturer's protocol.

### 2.2. Flow Cytometry

Mature seeds were extracted from individuals and stored in dry conditions at 4°C until use. The DNA content and reproductive pathway of JG2 and HD were determined by flow cytometric seed screen on 45 single seeds as described by Matzk et al. [39]. The flow cytometric seed screen is a simple and efficient method for identification of different reproductive modes in angiosperms depending on the proportional DNA content of the embryo and endosperm nuclei. The detailed procedure of flow cytometry analyses was presented by Galbraith [40]. In brief, the 15 seed samples were triturated in a Petri dish containing 1 mL of Galbraith's buffer supplemented with Triton-X-100 (0.1% *w*/*v*) to obtain a nuclear suspension. The nuclear suspension was filtered through a mesh of 40 *μ*m and incubated with 2.5 *μ*L of DNase-free RNase A (10 mg mL^−1^) on ice for 10 min. Samples were then stained by propidium iodide (50 *μ*g mL^−1^) after about 15 min of incubation on ice. The DNA content analyses were performed using an Accuri C6 flow cytometer (BD Biosciences, San Jose, CA, USA).

### 2.3. PacBio cDNA Library Construction and TGS

For cDNA preparation, a representative sample was produced by balanced mixes of RNA from 16 extractions (four cytotypes at four stages of development). Full-length cDNA was synthesized using the SMARTer™ PCR cDNA Synthesis Kit (TaKaRa Clontech Biotech, Dalian, China). The BluePippin™ Size Selection System (Sage Science, Beverly, MA, USA) was used to construct a size-fractionated library (0.5–6 kb). A total of two SMRT cells were sequenced on a Pacific Biosciences Sequel platform, producing 16.0 Gbp of raw reads. The sequencing data were uploaded to Sequence Read Archive (SRA) (https://www.ncbi.nlm.nih.gov/Traces/sra) with accession number SRP180032.

### 2.4. Illumina cDNA Library Construction and SGS

Equivalent amounts (mg) of RNA from each of the four extractions (a cytotype at four stages of development) were pooled to provide a sample for cDNA preparation for sequencing to generate a flower RNA-Seq dataset, and the four datasets from different cytotypes were used for TGS error correction. In addition, RNAs from flowers of different cytotypes at the functional megaspore-formed stage were used to construct cDNA libraries. SGS cDNA libraries were constructed using a NEBNext® Ultra™ RNA Library Prep Kit for Illumina® (NEB, Beverly, MA, USA). Qualified libraries were applied to NGS using an Illumina HiSeq™ 4000 platform (Illumina, San Diego, CA, USA). The raw-sequence read data were deposited in SRA with accession number SRP181246. High-throughput sequencing (both TGS and SGS) reported in this study was performed at Sagene Biotech Co. Ltd. (Guangzhou, China).

### 2.5. Iso-Seq Read Processing and Reference Transcriptome Reconstruction

SMART sequence data were compiled, processed employing SMRT Link 5.0.1 software. Initially, sequencing subread from BAM files can be combined into a circular consensus sequence (CCS). Secondly, the generated CCS reads were classified into full-length and non-full-length reads. The produced non-full-length and full-length FASTA files were then subjected to isoform-level clustering, followed by final Arrow polishing to obtain full-length consensus isoforms. All polished consensus reads were corrected using the four Illumina RNA-Seq flower data of differential cytotypes at four stages of development with the software Long-Read De Bruijn Graph Error Correction (LoRDEC) [41]. Finally, the consensus transcripts with high quality from each library were merged together, and redundancy was eliminated using the CD-HIT-EST tool (http://weizhongli-lab.org/cd-hit/) to obtain final FL isoforms. The longest transcript isoform in each cluster was selected as the representative unigene for further functional annotation [42].

### 2.6. Functional Annotation and Transcript Coding Prediction

All unigenes were aligned against NR (NCBI nonredundant protein sequence database), KOG (different protein and nucleotide databases of EuKaryotic Orthologous Groups), KEGG (Kyoto Encyclopedia of Genes and Genomes), Pfam (a database of conserved protein families or domains), and Swiss-Prot (a manually annotated, nonredundant protein database) by applying BLAST with an *E* value cutoff of 1 × 10^−5^. Blast2GO was used to retrieve associated Gene Ontology (GO) terms of unigenes based on BLASTX hit against NR database.

We used TransDecoder software to predict coding sequences of transcripts to gain high-quality sequences with reliable open reading frames. CPC [43] and CNCI [44] software with default parameters were then used to predict the remaining sequences. Each transcript was translated in all three possible frames, and any of the known protein family domains was identified through Pfam Scan in the Pfam database. The coding potential of transcripts would be excluded by Pfam Scan based on the Hidden Markov Model. The transcripts without coding potential were retained as lncRNAs for further analysis.

### 2.7. Differential Expression Analysis

Reads from cytotype JG2 and HD at the functional megaspore-formed stage were produced in this study. The data from cytotype JG1 and ZJJ at the functional megaspore-formed stage were derived from a previous study [25]. The trimmed reads of each cytotype were separately realigned to the high-quality reference transcriptome. The levels of unigene representation were measured via the FPKM method using Bowtie v2-2.l.0 by RSEM software [45]. Differential expression analysis of two cytotypes was checked using the edgeR package (https://www.r-project.org/), and the *P* values were adjusted using the Benjamini and Hochberg method. We set a fold change ≥ 2 and a corrected *P* value cutoff < 0.05 as the threshold for significant DEGs. DEGs were then subjected to test statistical enrichment in KEGG pathways and GO functions.

### 2.8. Real-Time PCR Validation

To verify our RNA-Seq results, 14 randomly selected DEGs were validated by real-time PCR. PCR primers are listed in [Supplementary-material supplementary-material-1]. We used a reverse transcription system (Fermentas, Burlington, Ontario, Canada) to reverse-transcribe RNA from each sample into cDNA. The iTaq™ Universal SYBR Green supermix (Bio-Rad) and iQ5 multicolour real-time PCR system (Bio-Rad, Hercules, CA, USA) were used to perform RT-qPCR. The relative expression levels for each gene were normalized, and cycle threshold values were calculated with the 2−*ΔΔ*CT method using the gene for EF1*α* as a housekeeping gene.

## 3. Results

### 3.1. DNA Content and Mode of Reproduction

**Figure 1:**
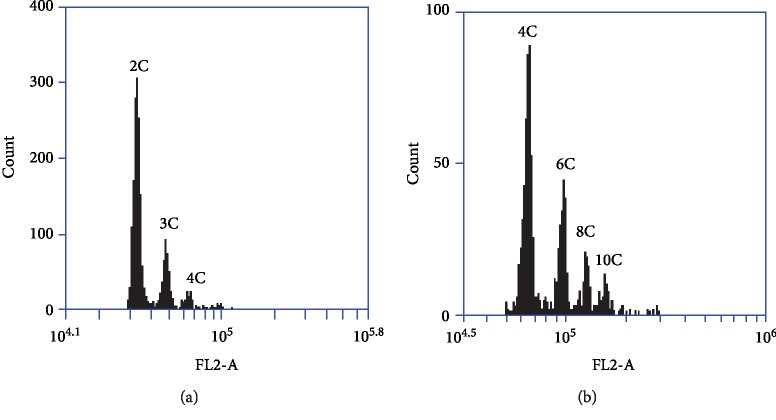
Uniparametric histogram of FL2-A fluorescence showing DNA content of nuclei from different cytotypes. (a) DNA content distribution in seeds of the cytotype JG2. (b) DNA content distribution in seeds of the cytotype HD. 2C, 3C, 4C, 6C, 8C, and 10C designate the appropriate *C* values for the individual peaks.

Based on the chromosome counting in root tips ([Supplementary-material supplementary-material-1]), JG2 and HD were confirmed to be diploid (2*n* = 2*x* = 28) and tetraploid (2*n* = 4*x* = 56), respectively. According to the flow cytometry screen, the seeds of the cytotype JG2 plant showed histograms with three clear peaks: 2C embryo and 3C+4C endosperm peaks (Figure 1(a)). In this case, the fluorescence values of the peaks were 32,270.3, 48,008.5, and 64,350.3. The distinct 4C peak was the evidence of an unreduced embryo sac, which indicated that two unreduced polar nuclei formed a 4C endosperm without fertilization. The 3C endosperm was generated after fusion of the two polar nuclei in reduced embryo sacs with one reduced male gamete. And the high 2C peak resulted from the autonomous development of the unreduced egg cell and the fertilization of the egg cell of reduced embryo sacs with reduced male gametes. Thus, the cytotype JG2 was classified as facultative autonomous apomixis, and the similar result of the flow cytometry screen was observed in *Arabis holboellii* [39]. The cytotype HD was defined as facultative autonomous/pseudogamous apomixis, when investigated seed samples yielded clear 4C, 6C, 8C, and 10C peaks (Figure 1(b)), which showed 4 : 6, 4 : 8, or 4 : 10 embryo : endosperm *C* values. The peak positions were values of 63,603.8, 95,388.7, 126,758.0, and 158,296.5. The 4C embryo was formed via fertilization of the reduced embryo sac and autonomous development of the unreduced egg cell. The 6C peak was produced after fusion of the two nuclei of the reduced embryo sac with one reduced male gamete. The 8C and 10C endosperms were derived through autonomous and pseudogamous development, respectively. Because only dry mature seeds were used, the detected peak could simply represent the G0/G1 phase cells, and endoreduplicated cells were not observed in *B*. *tricuspis* seeds.

### 3.2. A Full-Length Reference Transcriptome of the Female Flowers of *B. tricuspis*

**Figure 2:**
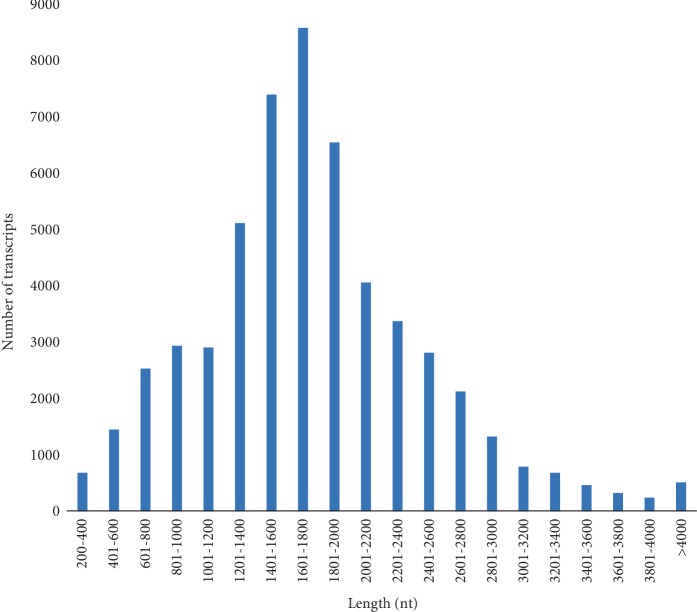
Length distribution of *B*. *tricuspis* transcripts.

To obtain a representative full-length floral transcriptome for *B*. *tricuspis*, female flowers were separately collected from four different cytotypes with contrasting reproductive modes and different ploidy levels: JG1 (2*n* = 2*x* = 28; obligate sexual), JG2 (2*n* = 2*x* = 28; facultative apomictic), ZJJ (2*n* = 3*x* = 42; obligate apomictic), and HD (2*n* = 4*x* = 56; facultative apomictic) (Table 1). After RNA extraction and library preparation, two SMRT cells were run on the PacBio Sequel system (Pacific Biosciences, Menlo Park, CA, USA) ([Supplementary-material supplementary-material-1]). Overall, 10,687,397 subreads were generated, with a total of 16,057,549,081 bases. The Iso-Seq information is presented in [Supplementary-material supplementary-material-1]. From these subreads, 812,517 consensus reads were generated and then divided into two classes (full-length and non-full-length reads). After polishing, 538,010 full-length nonchimeric reads were acquired, with a mean length of 1,781 bp. Subsequently, we followed error correction pipelines using the 45.69 Gb SGS clean bases, and redundancy was removed. Finally, 54,890 transcripts were obtained, with lengths ranging from 223 to 7,626 bp, N50 of 1,909 bp, and an average length of 1,771 bp with 46.55% GC content. In total, 50,220 transcripts (91.49%) were longer than 600 bp, and 44,374 (80.84%) were longer than 1 kb (Figure 2).

**Figure 3:**
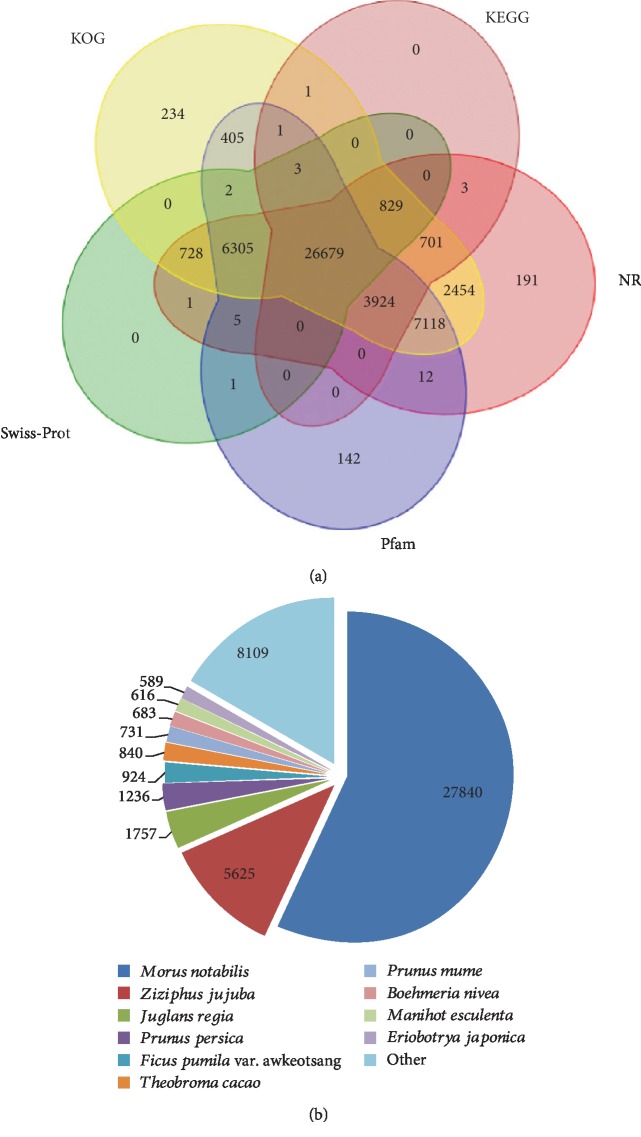
Functional annotation of the protein-coding transcripts of *B*. *tricuspis* using the public databases. (a) Venn diagram of the annotation between NR, Pfam, KOG, KEGG, and Swiss-Prot databases. (b) Distribution of homologous species annotated in the NR database.

Among the 54,890 transcripts, a total of 50,787 protein-coding transcripts were identified using TransDecoder (https://github.com/TransDecoder/TransDecoder/wiki). Of these, 49,739 transcripts were found in at least one of five databases: KOG, KEGG, Pfam, Swiss-Prot, and NR, and 26,679 high-scoring transcripts were identified in all five databases (Figure 3(a), [Supplementary-material supplementary-material-1]). Of the BLAST hits to the NR protein database, the transcripts had the highest number of hits to *Morus notabilis* (27,840 hits, 56.9%), followed by *Ziziphus jujuba* (5,625 hits, 11.5%) and *Juglans regia* (1,757 hits, 3.6%) proteins (Figure 3(b)). The Gene Ontology database was utilized to retrieve the GO identifiers for each unigene annotated in NCBI NR ([Supplementary-material supplementary-material-1]). Overall, 41,394 (82.0%) unigenes were assigned to 5,345 GO terms in three main categories: 2,743 biological process (BP), 1,885 molecular function (MF), and 717 different cellular components (CC). The results of KOG analysis showed that 49,384 annotated unigenes participated in 24 different KOG categories ([Supplementary-material supplementary-material-1]). Notably, 1,047 transcripts showed no obvious homologs anywhere in the public databases ([Supplementary-material supplementary-material-1]).

### 3.3. Expression Levels of Genes and Identification of Differentially Expressed Genes (DEGs)

**Figure 4:**
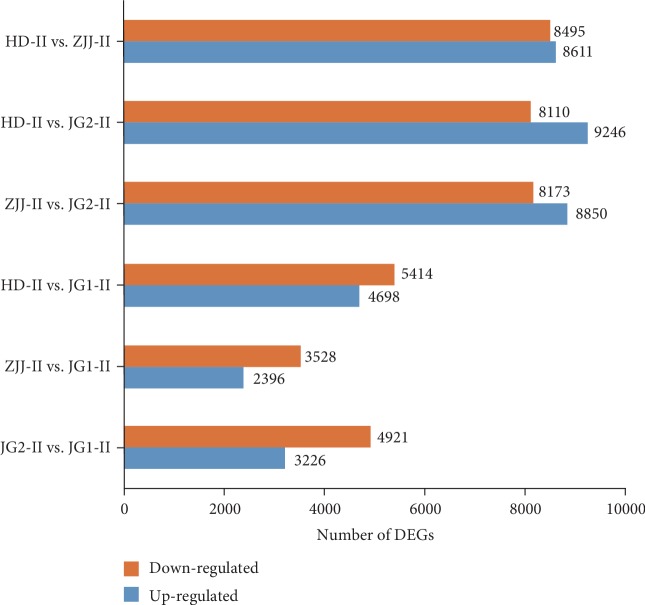
Number of differentially expressed genes (DEGs) by pairwise comparisons between different cytotypes.

In total, 7.04 × 10^8^ short reads were produced from 12 libraries at the functional megaspore-formed stage of the four cytotypes (JG1, JG2, ZJJ, and HD), with 6.98 × 10^8^ clean reads obtained for further analysis; each library approximately yielded 5.8 × 10^7^ clean reads ([Supplementary-material supplementary-material-1]). The percentage of Q20 bases was higher than 95.9% and indicated that high-quality raw reads were obtained from each library. After each reads were separately realigned to the high-quality reference transcriptome, the expression level of each transcript of the reference transcriptome was estimated in all cytotypes. DEGs were identified by pairwise comparisons between different cytotypes (Figure 4).

### 3.4. Validation of DEGs

RT-qPCR analysis was utilized to validate the results of DEGs obtained from the RNA-Seq data. Among the 14 DEGs tested, all but Unigene033992_01 displayed a similar expression pattern (up- or downregulation) in RT-qPCR assays as that obtained from RNA-Seq ([Supplementary-material supplementary-material-1] and [Supplementary-material supplementary-material-1]). A very good agreement between the two sets of results can be observed.

### 3.5. Genes Related to Apomeiosis

**Figure 5:**
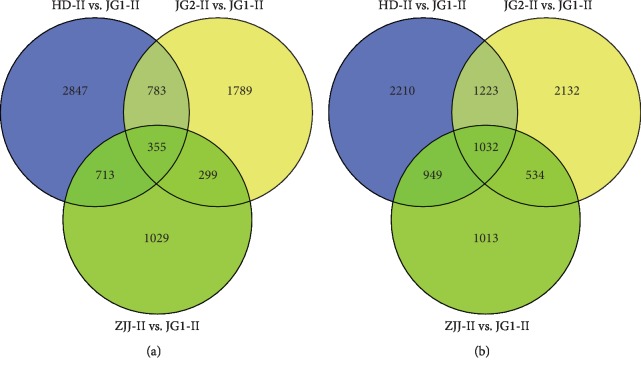
Venn diagrams showing the overlap of differentially expressed genes (DEGs) between different cytotypes. (a) Upregulated and (b) downregulated DEGs are shown.

**Figure 6:**
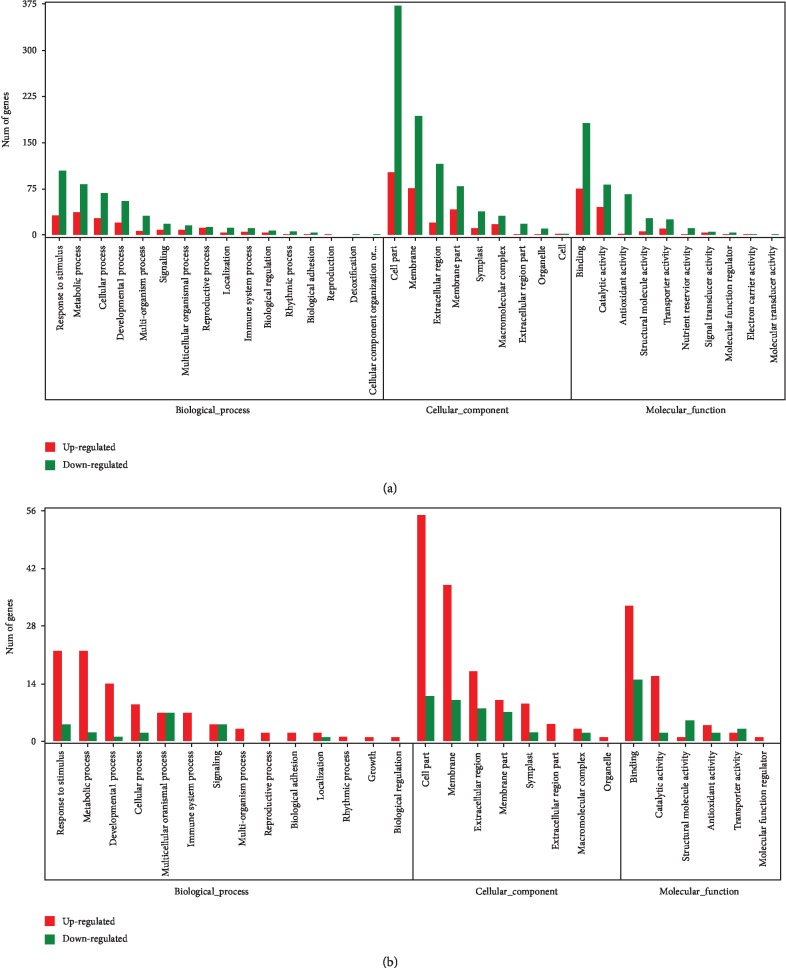
Classification of differentially expressed genes (DEGs) based on predicted Gene Ontology (GO) terms: (a) DEGs related to apomeiosis; (b) DEGs related to ploidy.

Venn diagrams were used to display list comparison between apomictic samples and a sexual sample. We identified that 355 genes were upregulated (Figure 5(a)) and 1,032 genes were downregulated (Figure 5(b)) during apomictic development in JG2-II vs. JG1-II, ZJJ-II vs. JG1-II, and HD-II vs. JG1-II. By excluding the effect of the differences in expression caused by ploidy levels, these DEGs were classified as genes related to apomeiosis, which were consistently differentially expressed between the apomictic and sexual cytotypes in this species independent of the ploidy level. GO assignments to classify these DEG functions (Figure 6(a)) indicated that the most enriched terms were “response to stimulus” (137 genes), “metabolic process” (121 genes), and “cellular process” (95 genes) in the BP category and “cell part” (474 genes), “membrane” (269 genes), and “extracellular region” (136 genes) in the CC category. Under MF category, “binding” (256 genes) was the most enriched, followed by “catalytic activity” (127 genes) and “antioxidant activity” (67 genes). Among the BPs enriched in this set, we could emphasise “reproductive process” and “reproduction” terms. This group contained 26 genes (13 upregulated and 13 downregulated) ([Supplementary-material supplementary-material-1]) of which eight are related to “cell division,” “asymmetric cell division,” “meiotic cell cycle,” “cell differentiation,” and “cell growth.” Notably, seven of these, including *structural maintenance of chromosomes protein 3*, *hypothetical protein L484_025226*, *zinc finger protein MAGPIE*, *serine/threonine-protein kinase TOUSLED*, *protein GRIP*, *receptor-like protein kinase FERONIA*, and *floral homeotic protein APETALA 2*, were upregulated in the apomictic ones.

To better understand transcriptional data in more detail, we carried out hierarchical clustering analysis of the 1,387 DEGs based on the Euclidean distance method ([Supplementary-material supplementary-material-1]). This analysis showed that the gene expression pattern of JG2-II was the most similar to that of HD-II, whereas the JG1-II gene expression pattern was the furthest from that of the other samples, suggesting the differential regulation of genes between the sexual and apomictic reproductive modes.

### 3.6. Genes Related to Ploidy

Overall, 217 DEGs were identified in ZJJ vs. JG1, HD vs. JG1, ZJJ vs. JG2, HD vs. JG2, and HD vs. ZJJ, including 164 overexpressed (i.e., expression level increased with ploidy level) and 53 underexpressed genes (i.e., expression level decreased as ploidy level increased). These DEGs corresponded to potential genes related to ploidy independent of the reproductive mode in *B*. *tricuspis*. The enriched GO terms of these genes represented 14 BP, 6 MF, and 8 CC terms (Figure 6(b)). In the BP category, the most enriched terms were “response to stimulus” (26 genes), “metabolic process” (24 genes), and “developmental process” (15 genes). For the CC category, the highest scores were recorded for GO terms related to “cell part” (66 genes), “membrane” (48 genes), and “extracellular region” (25 genes). Under the MF category, “binding” (48 genes) was the most enriched, followed by “catalytic activity” (18 genes) and “antioxidant activity” (6 genes).

### 3.7. Ploidy vs. Reproductive Mode of Gene Expression Control

Venn diagrams ([Supplementary-material supplementary-material-1]) allowed the identification of nine genes associated with both apomixis and ploidy (Table 2). Of these, three genes were mainly involved in response to abiotic and biotic stimulus, and the other genes corresponded to *metal tolerance protein 5*, *metallothionein-like protein 1*, *endoplasmin-like protein*, *1-aminocyclopropane-1-carboxylate oxidase homolog 4-like*, *ubiquitin carboxyl-terminal hydrolase 23*, and *an unknown protein*.

## 4. Discussion

From the viewpoint of reproductive biology, apomictic *B*. *tricuspis* constitutes a diplosporous species whereby the MMC develops directly to a functional megaspore, completely skipping the process of meiosis, and then gives rise to a functional, unreduced embryo sac. In the present study, we described a full-length reference floral transcriptome of *B*. *tricuspis* along with expression analysis on flowers collected from cytotypes exhibiting different ploidy (2*x*, 3*x*, and 4*x*) and/or reproductive forms at the functional megaspore-formed stage. This led to the identification of genes that show altered expression profiles in response to changes in the reproductive mode (apomictic to sexual) and ploidy.

### 4.1. Transcriptome Signatures of Apomeiosis Processes in Diplosporous *B. tricuspis*

At the functional megaspore-formed stage, apomictic cytotypes encompass the apomeiotic program and induction of diplosporous embryo sac development. Excluding the effect of the differences in expression caused by ploidy levels, we identified 1,387 DEGs associated with apomeiosis. Notably, over 74% of DEGs were downregulated in diplosporous samples and furthermore showed a general pattern of downregulation in apomictic cytotypes for most GO terms (Figure 6). A similar result was observed in the diplosporous *Boechera divaricarpa* [15], which was hypothesized to reflect the differing reproductive modes (e.g., apomeiosis versus meiosis). Additionally, the studies in aposporous *Hypericum perforatum* also showed the global downregulation of expression of numerous genes compared to sexual accession during the early stages of ovule development [27]. This result exhibits an amazing similarity in whole-transcriptome changes prior to somatic cell development, with unreduced functional megaspores developing to an embryo sac in both aposporous and diplosporous species.

Through functional classification of DEGs related to apomeiosis, we identified several classes of annotation directly associated with developmental process, signalling, reproductive process, biological regulation, rhythmic process, and reproduction. In reproductive process and reproduction terms, seven gene products involved in “cell division,” “asymmetric cell division,” “meiotic cell cycle,” “cell differentiation,” and “cell growth” were upregulated, which may reflect ameiotic processes. The data indicated that reproductive genes, especially those related to cell differentiation and cell cycle process, comprised significant factors for regulating apomeiosis which is in agreement with previous findings in Arabidopsis [46–49] and maize [50–52].

Moreover, 121 DEGs (38 upregulated and 83 downregulated) were involved in “metabolic processes” (i.e., glucose metabolic process, fatty acid biosynthetic process) related to the generation or catabolism of precursor metabolites and energy, and 95 DEGs (27 upregulated and 68 downregulated) were involved in “cellular processes” (i.e., protein folding, cell wall organisation, or biogenesis) (Figure 6). Similarly, modulation of genes involved in carbohydrate and lipid metabolism between sexual and apomictic plants was reported by Schmidt et al. [53] and Galla et al. [27, 54]. These results may suggest that different overall energy and material were consumed in the ameiotic compared to the meiotic process, as the MMC develops directly to a functional megaspore, completely omitting meiosis.

We also found that 137 DEGs (32 upregulated and 105 downregulated) were involved in “response to stimulus,” which indicated that sexual and apomictic reproduction were affected by great different biotic or abiotic stress. In the multicellular green alga *Volvox carteri*, which can reproduce both sexually and asexually, stress and reactive oxygen species-induced DNA damage plays an important role in determining which reproductive mode is selected [55]. In plants, numerous experimental studies in facultative apomictic accessions have provided evidence that stresses such as salt stress, photoperiod extension, and water deficit lead to an increase in sexual embryo sac frequencies compared to those in apomeiotic accessions [56–58]. Several previous transcriptome studies have also reported the inclusion of biotic and abiotic stress responses in the differential expression list during aposporous [27, 28] and diplosporous development [59]. In addition, in *Citrus*, which is sporophytic apomictic, genes associated with stress response are also detected in transcriptome comparisons of sexual and nucellar embryos [60]. Furthermore, the deregulation of stress response genes might be important in the preparation for apomeiosis transition in *Boechera* [61]. In particular, Hörandl and Hadacek [62] proposed a hypothesis indicating the participation of stress response pathways in meiosis initiation; more recently, Hörandl and Speijer proposed an “oxidative stress initiation” model describing how endogenous oxidative stress could trigger sex and drive the evolution of meiotic sex [63]. In the present research, we identified abundant DEGs related to the response to abiotic and biotic stress; these findings thus serve as a valuable resource for the “oxidative stress initiation” model.

### 4.2. Correlations of Polyploidy and Apomixis in *B. tricuspis*

In plants, unlike earlier reports, recent studies provide evidence that apomixis is not necessarily exclusively and functionally connected with polyploidy [15, 17]. In particular, natural [17, 64] and experimental [65] apomictic diploids have been observed and produced, respectively. In the present study, we also exploited the rare phenomenon of diploid gametophytic apomixis in *B*. *tricuspis*, a species that offers a suitable system for polyploidy and apomixis research owing to availability of different ploidy levels and reproductive modes. In *B*. *tricuspis*, we could compare gene expression of the different ploidy levels (2*x*, 3*x*, and 4*x*) irrespective of the reproductive mode to examine the effects of polyploidization. Pairwise comparisons allowed the identification of 217 candidate genes (75.6% upregulated; 24.4% downregulated) whose expression was controlled by changes of the ploidy level in the female inflorescences. Polyploidy exerts considerable effect on duplicate gene expression, including silencing and up- or downregulation of one of the duplicated genes [66, 67]. In *B*. *tricuspis*, it appeared that a larger percentage of genes showed upregulation of expression along with incremental doses of genome. Functional classification further showed that DEGs related to ploidy were significantly enriched for processes as “response to stimulus” (i.e., hydrogen peroxide, salt stress, and water deprivation), “metabolic process” (i.e., carbon fixation, fatty acid biosynthetic process, and lysine biosynthesis), and “developmental process” (i.e., embryo development and plant ovule development). The results are consistent with the morphology observation, wherein polyploidy individuals exhibited high seed set and vegetable growth compared to those of diploid individuals in the species.

In order to examine the extent of correlation between polyploidy and apomixis, we generated Venn diagrams, from which nine DEGs associated with both apomixis and ploidy were detected, with the majority being related to response to stress. In particular, a key gene encoding a “ubiquitin carboxyl-terminal hydrolase” protein participated in ubiquitin-mediated protein degradation. The ubiquitin-mediated pathway has been reported to directly and indirectly regulate the organisation of microtubular spindles along with nucleus positioning and identity in developing maize embryo sacs [68], and studies in *Hieracium praealtum* [69] and *Hypericum perforatum* [70] have revealed that ubiquitin proteasome components are enriched in aposporous initial cells. These previous findings indicated that at least some genes related to ploidy might be involved in diplosporous development and also provided preliminary molecular evidence for the function of polyploidization with regard to apomixis. Polyploidization could result in chromosome duplications and rearrangements that cause the drastic changes of gene expression at the transcriptional level and posttranscriptional level. The great changes provide a possible route to induce apomixis through deregulation of the sexual development pathway such that the functional megaspore develops without meiosis and embryo develops without fertilization [71]. Although hybridization in diploids has the similar influence on inducing apomixis, the significantly lower tolerance of DNA damage and deleterious mutations [72, 73] makes apomictic diploids rare in nature. Our results also suggested that expression of apomixis in *B*. *tricuspis* is not restricted to polyploids, and apomixis may rely on the deregulation of the sexual pathway.

## 5. Conclusions

The present study generated a full-length reference floral transcriptome of *B*. *tricuspis* and identified candidate genes related to apomeiosis and ploidy. This evidence provided clues regarding the molecular pathways involved in diplosporic apomeiosis and ploidy. Nevertheless, further research concerning genomic and functional characterization is needed to reveal the nature of the associated genetic determinants.

## Figures and Tables

**Table 1:** Information on the *B*. *tricuspis* cytotypes used for flower transcriptome analyses.

Cytotype	Ploidy	Reproduction	Flower reproductive morphology
JG1^a^	2*n* = 2*x*	Sexual	Monoecious
JG2	2*n* = 2*x*	Facultative apomictic	Gynoecious
ZJJ^b^	2*n* = 3*x*	Obligate apomictic	Gynoecious
HD	2*n* = 4*x*	Facultative apomictic	Monoecious

^a^JG1 and ^b^ZJJ were used in a previous study [26], named “S” and “A”, respectively.

**Table 2:** Genes associated with both apomixis and ploidy.

Gene ID	RNA-Seq (FPKM value)				Description	GO term
	JG1-II	JG2-II	ZJJ-II	HD-II		
Unigene001469_01	0	0.67	5.02	24.04	Metal tolerance protein 5	GO:0010043//response to zinc ion
Unigene002044_01	0	19.31	209.00	484.59	Metallothionein-like protein 1	—
Unigene035240_01	0.04	7.24	27.28	49.40	Endoplasmin-like protein	—
Unigene035398_01	0	1.17	20.37	38.12	1-Aminocyclopropane-1-carboxylate oxidase homolog 4-like	—
Unigene042048_01	0.44	4.09	19.81	31.60	Hypothetical protein L484_018886	GO:0050896//response to stimulus
Unigene044889_01	663.49	13.5	1.83	0.19	—	—
Unigene045442_01	0	0.59	5.30	9.67	Uncharacterized protein LOC18767759	GO:0050896//response to stimulus
Unigene048669_01	0.04	1.08	12.00	21.13	Phospho-2-dehydro-3-deoxyheptonate aldolase 1	GO:0050896//response to stimulus
Unigene049873_01	76.75	6.54	2.23	0	Ubiquitin carboxyl-terminal hydrolase 23	—

## Data Availability

The raw data are available in the NCBI Sequence Read Archive (SRA) repository with identifiers SRP180032 and SRP181246. Voucher specimens of the plant material are deposited at the National Field Genebank for Ramie of China, Changsha, Hunan, China, under the following deposition numbers: JG1 (No. ZM 1536), JG2 (No. ZM 1826), ZJJ (No. ZM 1900), and HD (No. ZM 1522).

## Supplementary Materials

Supplementary Materials Table S1: calendar of the reproductive development of B. tricuspis. Table S2: list of primer sequences used for real-time PCR analysis. Table S3: PacBio SMRT sequencing information of the flowers of B. tricuspis. Table S4: summary of B. tricuspis Iso-Seq. Table S5: functional annotation of protein-coding transcripts of B. tricuspis. Table S6: Illumina sequencing information of samples at the functional megaspore-formed stage of the four cytotypes. Table S7: the differentially expressed genes used for quantitative real-time PCR. Table S8: genes related to apomeiosis in “reproductive process” and “reproduction” terms. Figure S1: microphotographs showing root tip cells of B. tricuspis cytotypes at metaphase: (a) chromosome figures of JG2 (2*n* = 28); (b) chromosome figures of HD (2*n* = 56). Figure S2: analysis of functional categories of protein-coding transcripts of B. tricuspis. (a) Gene Ontology classification of protein-coding transcripts. (b) KOG functional classification of protein-coding transcripts. Figure S3: quantitative real-time PCR results for differentially expressed genes. Figure S4: clustering analysis of differentially expressed genes related to apomeiosis. Figure S5: Venn diagrams showing nine genes associated with both apomixis and ploidy.
