# Supplementary material for: A Full-Length Reference Floral Transcriptome of Boehmeria tricuspis Provides Insights into Apomeiosis and Polyploidy
Source: Int J Genomics. 2019 Dec 16;2019:4025747. doi: 10.1155/2019/4025747 (PMC6948294; doi:10.1155/2019/4025747)
Supplement: Supplementary Materials — Table S1: calendar of the reproductive development of B. tricuspis. Table S2: list of primer sequences used for real-time PCR analysis. Table S3: PacBio SMRT sequencing information of the flowers of B. tricuspis. Table S4: summary of B. tricuspis Iso-Seq. Table S5: functional annotation of protein-coding transcripts of B. tricuspis. Table S6: Illumina sequencing information of samples at the functional megaspore-formed stage of the four cytotypes. Table S7: the differentially expressed genes used for quantitative real-time PCR. Table S8: genes related to apomeiosis in “reproductive process” and “reproduction” terms. Figure S1: microphotographs showing root tip cells of B. tricuspis cytotypes at metaphase: (a) chromosome figures of JG2 (2n = 28); (b) chromosome figures of HD (2n = 56). Figure S2: analysis of functional categories of protein-coding transcripts of B. tricuspis. (a) Gene Ontology classification of protein-coding transcripts. (b) KOG functional classification of protein-coding transcripts. Figure S3: quantitative real-time PCR results for differentially expressed genes. Figure S4: clustering analysis of differentially expressed genes related to apomeiosis. Figure S5: Venn diagrams showing nine genes associated with both apomixis and ploidy. [file 4025747.f1.pdf]

Table S1: Calendar of the reproductive development of *B. tricuspis*.

Cytotype	Stage[a]	Ovule development	Femal glomerule diameter [mm]	Browning rate of exserted stigma [%]
Apomictic	I	MMC formed	1.5	≤20
	II	Functional megaspores formed (apomeiosis)	1.8	21-40
	III	Mature embryo sac formation	2.5	41-90
	IV	Mature embryo formation	3.0	≥91
Sexual	I	MMC formed	1.6	≤30
	II	Functional megaspores formed (tetrad to degeneration of megaspores)	1.9	31-50
	III	Mature embryo sac formation	2.4	51-90
	IV	Mature embryo formation	2.9	≥91

[a] according to Q.T. et al. (2017)

Table S2: List of primers sequences used for real time PCR analysis.

Gene ID	Forward Primer	Reverse Primer
Unigene042048_01	ACAAGCCAATCCCACCAATC	TTCTCTTCCTTGCCAGTGTACC
Unigene001412_01	GAAGCTCATGAGGAAGTTCGTG	AATGGTCTTGGGCTTCTTGG
Unigene004190_01	TCGGAAGGAGATGGACTTTTGG	ACCGTCCATCTCGTACATCTTG
Unigene005022_01	CTAGAATTGCCGTAGGAAGT	CCGGCGAAGACGAAGATAA
Unigene010575_01	CGTTGGCTTCTTGCTTCTCTTC	AGTGGAAACCAACGCTCTTG
Unigene005530_01	AGTGAGTGAGTGAGCATGGC	CTGTAGCCCTCGACGAATCC
Unigene033992_01	ATGCTGCGAGTTCAAAACCG	TGTGGTGCATGTATCTGTGC
Unigene004215_02	AGATTGGTTGCTGCCGAATC	ACAGTCTGCACCGCAAAATC
Unigene016354_01	GCATGTCTCATCATCATCACG	AGCATCTCTTCTCAGTCCT
Unigene025939_01	TGATCATGGCCATTCGATCG	CAAAAACATGCACGCCCATG
Unigene010179	GGAGCTGGAGGTAGGATG	TGATCTGGCTTAGGGTG

Unigene_01		AAT
Unigene024393_01	GTTCCTCGCAACACACACAC	AGAACGTCCACGAAGCCATT
Unigene006965_01	TTCTCTCCATTCGCTTCTACG	ACAAATCTTCTCATCTGCCG
Unigene005553_01	CATGCTTCTGCTCCTACCTAA	GCAGAGGCGACTTTCTTG
EF1α	ATCAAGCCCACTAAGCCCTT	TTCTCTCTGGCAGTGACCTC

Table S3: PacBio SMRT sequencing information of the flowers of *B. tricuspis*.

Cell Name	Number of Subreads	Subreads [nt]	Mean Length of Subreads [nt]	Number of CCS Reads	CCS [nt]	Mean CCS Read Length [nt]
BT-F1	6759718	10079763373	1491	501296	932016199	1859
BT-F2	3927679	5977785708	1521	311221	576273329	1851

Table S4: Summary of *B. tricuspis* IsoSeq.

Number of Consensus Reads	Number of Five Prime Reads	Number of Three Prime Reads	Number of Poly-A Reads	Number of Non-full-length Reads	Number of Full-length Reads	Number of Full-length non-chimeric Reads	Mean Full-length Non-chimeric Read Length [nt]
812517	649739	640935	631391	235773	561190	538010	1781

Table S5: Functional annotation of protein-coding transcripts of *B. tricuspis*.

Total	NR	Swissport	KEGG	KOG	Pfam	Annotated	Without Annotation
50787	48950	34553	32141	49384	44597	49739	1047

Table S6: Illumina sequencing information of samples at the functional megaspore-formed stage of the four cytotypes.

Sample	Raw Bases	Raw Reads	Clean Bases [%]	Clean Reads [%]	Clean Q20 [%]

JG1-II-1	7,926,329,700	52,842,198	7,914,718,954 (99.85%)	52,792,902 (99.91%)	7,597,262,480 (95.99%)
JG1-II-2	7,787,846,400	51,918,976	7,776,844,314 (99.86%)	51,870,282 (99.91%)	7,461,983,503 (95.95%)
JG1-II-3	7,944,276,600	52,961,844	7,932,618,602 (99.85%)	52,916,390 (99.91%)	7,610,075,065 (95.93%)
JG2-II-1	7,473,756,300	49,825,042	7,337,346,518 (98.17%)	48,956,560 (98.26%)	7,077,062,487 (96.45%)
JG2-II-2	8,308,687,500	55,391,250	8,183,236,174 (98.49%)	54,572,418 (98.52%)	7,940,420,808 (97.03%)
JG2-II-3	9,534,023,700	63,560,158	9,376,464,260 (98.35%)	62,540,714 (98.40%)	9,058,958,222 (96.61%)
ZJJ-II-1	10,796,442,900	71,976,286	10,787,279,944 (99.92%)	71,942,154 (99.95%)	10,546,917,977 (97.77%)
ZJJ-II-2	10,848,476,700	72,323,178	10,838,625,166 (99.91%)	72,286,118 (99.95%)	10,591,391,691 (97.72%)
ZJJ-II-3	9,551,915,100	63,679,434	9,543,251,564 (99.91%)	63,646,006 (99.95%)	9,328,710,221 (97.75%)
HD-II-1	8,870,079,000	59,133,860	8,723,734,448 (98.35%)	58,173,248 (98.38%)	8,428,033,398 (96.61%)
HD-II-2	8,167,272,600	54,448,484	8,053,196,360 (98.60%)	53,702,790 (98.63%)	7,819,937,094 (97.10%)
HD-II-3	8,322,535,500	55,483,570	8,191,297,670 (98.42%)	54,621,928 (98.45%)	7,946,620,567 (97.01%)

Table S7: The differentially expressed genes using for quantitative real-time PCR.

Gene ID	RNA-Seq (FPKM Value)				Description
	JG1-II	JG2-II	ZJJ-II	HD-II	
Unigene042048_01	0.47	4.09	19.81	31.6	hypothetical protein L484_018886
Unigene001412_01	21.72	174.91	601.01	745.81	PREDICTED: 17.9 kDa class II heat shock protein-like
Unigene004190_01	2.89	169.20	37.09	92.12	Calcium-binding protein CML38
Unigene005022_01	10.33	82.88	410.90	117.40	PREDICTED: aquaporin TIP1-1-like
Unigene010575_01	2.47	55.55	165.39	228.90	PREDICTED: uncharacterized protein LOC103954024
Unigene005530_01	9.89	230.03	485.78	80.94	Chlorophyll a-b binding protein 151
Unigene033992_01	0.41	22.15	22.13	28.11	Sphingoid base hydroxylase 2

Unigene004215_02	1326.52	0	0.66	3.93	PREDICTED: protein CHROMATIN REMODELING 35-like isoform X3
Unigene016354_01	92.18	0.04	18.21	0.65	Mlo-related protein
Unigene025939_01	287.47	0	0.14	0.03	PREDICTED: dormancy-associated protein homolog 3
Unigene010179_01	55.89	0.10	0	0	Omega-6 fatty acid desaturase, endoplasmic reticulum isozyme 2
Unigene024393_01	1.58	3.00	12.10	19.19	Peroxisomal membrane protein
Unigene006965_01	139.19	93.39	11.03	0.11	PREDICTED: F-box protein GID2-like
Unigene005553_01	579.41	864.09	111.48	38.67	hypothetical protein L484_027077

Table S8: Genes related to apomeiosis in “reproductive process” and “reproduction” terms.

Gene ID	RNA-Seq (FPKM Value)				Description	GO Term
	JG1-II	JG2-II	ZJJ-II	HD-II		
Up-regulated						
Unigene004443_01	0	4.97	12.57	8.28	Myb family transcription factor APL	GO:0009566//fertilization
Unigene004656_01	0	20.61	19.74	8.18	uncharacterized protein LOC100265927	GO:0022414//reproductive process
Unigene006134_02	0	5.95	10.52	13.64	structural maintenance of chromosomes protein 3	GO:0051301//cell division
Unigene007852_01	0	23.25	66.42	28.05	splicing factor U2af small subunit B	GO:0048573//photoperiodism, flowering
Unigene011378_01	0	2.94	1.61	2.84	hypothetical protein L484_025226	GO:0009825//multidimensional cell growth
Unigene014383_01	0	10.28	7.59	8.14	Zinc finger protein MAGPIE	GO:0008356//asymmetric cell division
Unigene019135_01	0	7.71	2.72	9.81	Pyruvate kinase isozyme A	GO:0048609//multicellular organismal reproductive process

Unigene022271_01	0	16.99	13.32	14.29	mitogen activated protein kinase	GO:0010229//inflorescence development
Unigene033127_01	0	5.63	8.81	7.33	Serine/threonine-protein kinase TOUSLED	GO:0051301//cell division
Unigene034760_01	0	0.27	1.06	5.11	Protein GRIP	GO:1903046//meiotic cell cycle process
Unigene038297_01	0	23.66	18.66	4.10	putative nucleoredoxin 1	GO:0009860//pollen tube growth
Unigene023755_01	0.06	8.91	16.25	21.72	Receptor-like protein kinase FERONIA	GO:0030308//negative regulation of cell growth
Unigene045567_01	0	2.12	9.39	0.76	Floral homeotic protein APETALA 2	GO:0030154//cell differentiation
Down-regulated						
Unigene006953_01	24.55	0	0	0	11S globulin precursor isoform 1B	GO:0048609//multicellular organismal reproductive process
Unigene011564_01	20.37	0	0	0	CCG-binding protein 1	GO:0022414//reproductive process
Unigene019776_02	33.65	0.49	0.43	0.04	hypothetical protein L484_021422	GO:0022414//reproductive process
Unigene021325_01	30.57	0.03	0.11	0	oxysterol-binding protein-related protein 3A	GO:0009566//fertilization
Unigene021971_01	8.97	0	0	0	WD repeat-containing protein LWD1-like	GO:0048573//photoperiodism, flowering
Unigene028099_01	11.59	0	0	0	11S globulin precursor isoform 1B	GO:0048609//multicellular organismal reproductive process
Unigene028918_01	4.24	0	0	0	11S globulin precursor isoform 1B	GO:0048609//multicellular organismal reproductive process
Unigene029815_01	41.66	0	0	0	putative nucleoredoxin 1	GO:0009860//pollen tube growth
Unigene030946_01	4.14	0	0	0	11S globulin precursor isoform 1B	GO:0048609//multicellular organismal reproductive process
Unigene034776_01	31.34	0.10	0	0	11S globulin precursor isoform 1B	GO:0048609//multicellular organismal reproductive process
Unigene03	1.9	0	0	0	receptor-like protein	GO:0030308//negativ

6981_01	3				kinase FERONIA	e regulation of cell growth
Unigene037390_01	2.61	0	0	0	11S globulin precursor isoform 1B	GO:0048609//multicellular organismal reproductive process
Unigene040071_01	13.92	1.90	0	0.59	hypothetical protein L484_010548	GO:0048573//photoperiodism, flowering

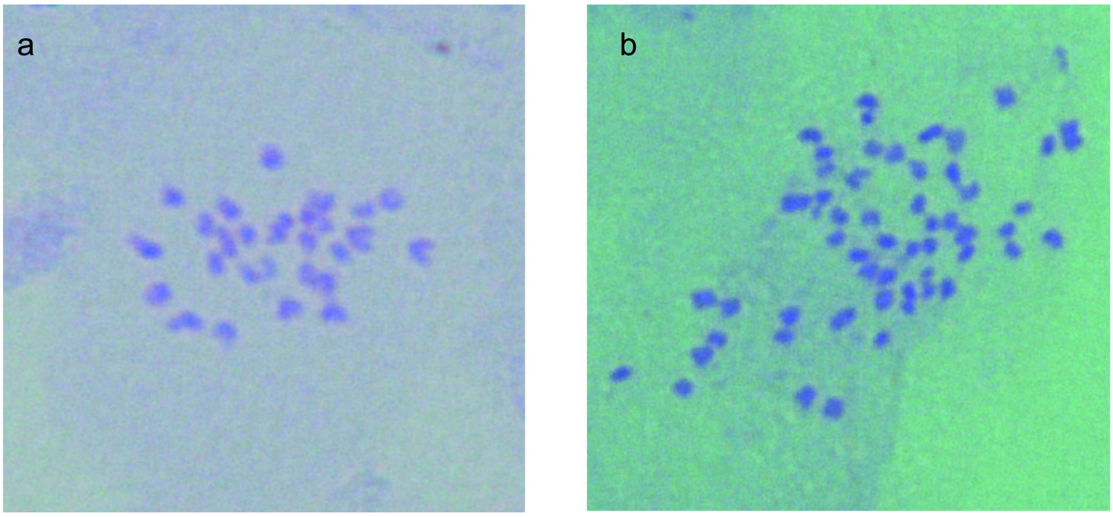

Figure S1: Microphtographs showing root tip cells of *B. tricuspis* cytotypes at metaphase: (a) Chromosome figures of JG2 (2*n*=28). (b) Chromosome figures of HD (2*n*=56).

a

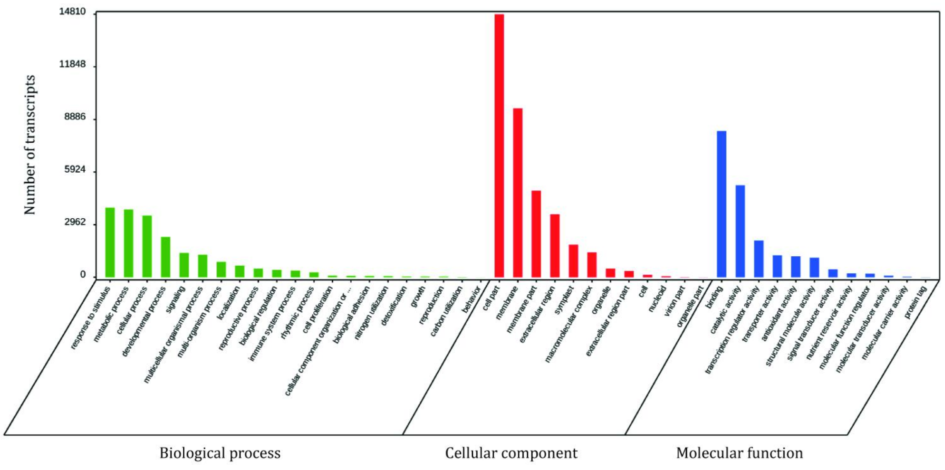

b

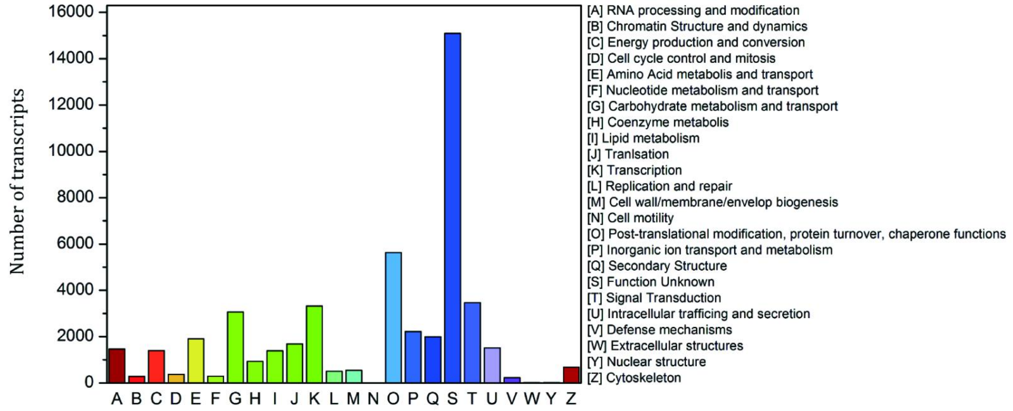

Figure S2: Analysis of functional categories of protein-coding transcripts of *B*. *tricuspis*. (a) Gene ontology classification of protein-coding transcripts. (b) KOG functional classification of protein-coding transcripts.

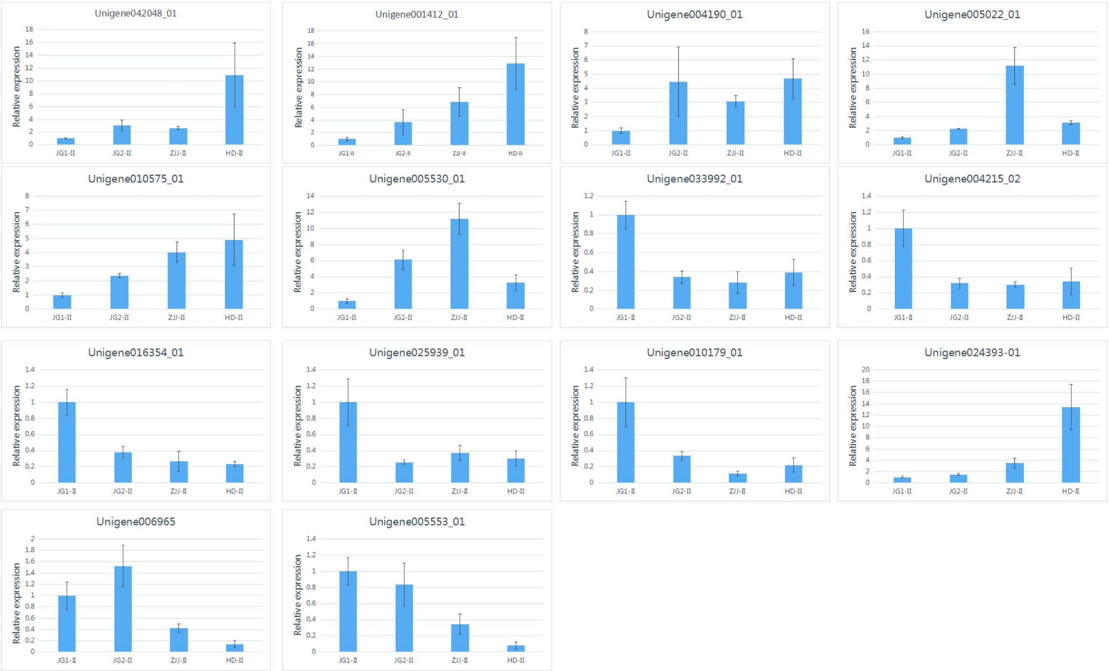

Figure S3: Quantitative real-time PCR results for differentially expressed genes.

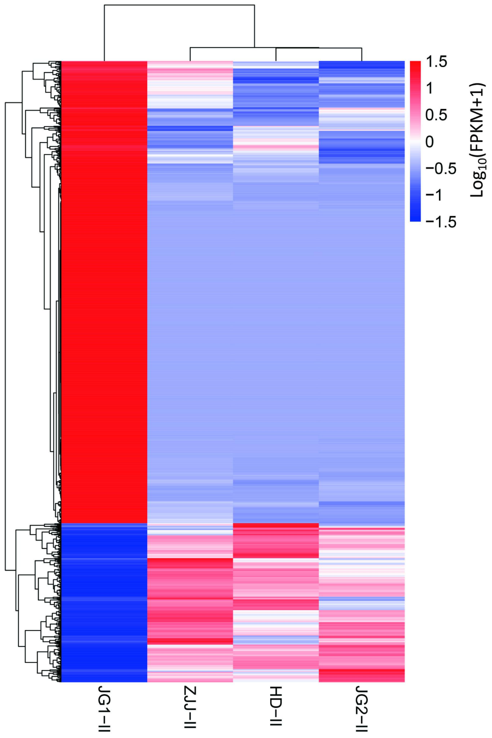

Figure S4: Clustering analysis of differentially expressed genes related to apomeiosis.

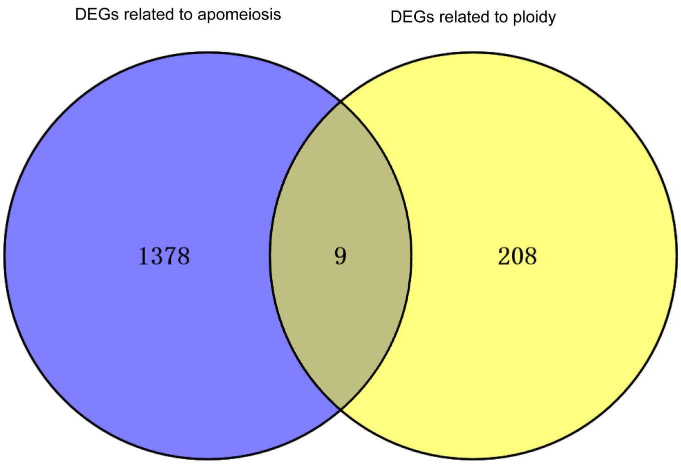

Figure S5: Venn diagrams showing nine genes associated with both apomixis and ploidy.
